# Nanocrystalline Cellulose as a Versatile Engineering Material for Extrusion-Based Bioprinting

**DOI:** 10.3390/pharmaceutics15102432

**Published:** 2023-10-07

**Authors:** Sophia A. Read, Chee Shuen Go, Miguel J. S. Ferreira, Cosimo Ligorio, Susan J. Kimber, Ahu G. Dumanli, Marco A. N. Domingos

**Affiliations:** 1 Department of Mechanical, Aerospace and Civil Engineering, School of Engineering, Faculty of Science and Engineering & Henry Royce Institute, The University of Manchester, Manchester M13 9PL, UK; sophia.read-2@postgrad.manchester.ac.uk (S.A.R.); chee.go@student.manchester.ac.uk (C.S.G.); miguel.ferreira@manchester.ac.uk (M.J.S.F.); 2 Department of Materials, School of Natural Sciences, Faculty of Science and Engineering & Henry Royce Institute, The University of Manchester, Manchester M13 9PL, UK; cosimo.ligorio@postgrad.manchester.ac.uk (C.L.); ahugumrah.parry@manchester.ac.uk (A.G.D.); 3 Division of Cell Matrix Biology and Regenerative Medicine, School of Biological Sciences, Faculty of Biology, Medicine and Health, The University of Manchester, Manchester M13 9PT, UK

**Keywords:** 3D bioprinting, tissue engineering, biomaterials, alginate, cellulose nanocrystals, bioinks

## Abstract

Naturally derived polysaccharide-based hydrogels, such as alginate, are frequently used in the design of bioinks for 3D bioprinting. Traditionally, the formulation of such bioinks requires the use of pre-reticulated materials with low viscosities, which favour cell viability but can negatively influence the resolution and shape fidelity of the printed constructs. In this work, we propose the use of cellulose nanocrystals (CNCs) as a rheological modifier to improve the printability of alginate-based bioinks whilst ensuring a high viability of encapsulated cells. Through rheological analysis, we demonstrate that the addition of CNCs (1% and 2% (*w*/*v*)) to alginate hydrogels (1% (*w*/*v*)) improves shear-thinning behaviour and mechanical stability, resulting in the high-fidelity printing of constructs with superior resolution. Importantly, LIVE/DEAD results confirm that the presence of CNCs does not seem to affect the health of immortalised chondrocytes (TC28a2) that remain viable over a period of seven days post-encapsulation. Taken together, our results indicate a favourable effect of the CNCs on the rheological and biocompatibility properties of alginate hydrogels, opening up new perspectives for the application of CNCs in the formulation of bioinks for extrusion-based bioprinting.

## 1. Introduction

Polymeric hydrogels, both natural and synthetic, are traditionally employed in the biomedical field to create three-dimensional (3D) systems capable of mimicking the native extracellular matrix (ECM) microenvironment and supporting the function of mammalian cells in vitro. Besides providing structural support, these systems can also be designed with tailored physicochemical properties, which are essential in regulating tissue-specific morphogenesis and homeostasis. More recently, polymeric hydrogels have also found application in the formulation of biological inks (i.e., bioinks) through the combination of cells with biomaterials that can be shaped into 3D artificial tissues via additive manufacturing (AM) in a process known as ‘3D bioprinting’ [1].

Despite sharing the same principles, not all AM processes are amenable to the processing of living cells, thus making them unsuitable for bioprinting. Current available techniques are based on either vat photopolymerisation, material jetting or extrusion principles, with the latter being by far the most exploited in tissue engineering and regenerative medicine (TE&RM). Ideally in extrusion-based bioprinting, a polymeric ink should flow easily through the nozzle without clogging, and quickly recover its shape after printing and crosslinking—all without negatively affecting cell viability. This process requires formulating polymeric inks with suitable rheological properties, including shear thinning, viscosity, yield stress, and recovery. These can be fine-tuned using different strategies that are available for this specific purpose, which are reviewed in detail elsewhere [2]. Whilst manipulating the rheological properties of hydrogels to improve shape fidelity or cell viability independently to one another has become common practice, striking a balance between these two important, yet often opposing, requirements still proves difficult. For example, while increasing the polymer concentration or molecular weight to obtain high-viscosity inks can improve shape fidelity post-printing, biological indicators such as cell viability, proliferation, and differentiation are often reduced as a consequence of the increased stiffness of these constructs.

Clearly, attaining both the printing and biological requirements is a bottleneck in 3D bioprinting that often forces the establishment of a compromise between contesting elements of material biomimicry and processability, commonly referred to as the ‘biofabrication window’. As reported by Schwab et al., the traditional fabrication window is relatively narrow and does not necessarily reflect the optimal conditions for bioprinting [3]. Novel strategies based on the use of suspension baths have recently been proposed to expand the range of processable inks with high shape fidelity and without compromising cell viability. In some cases, these can represent an alternative to the addition of viscosity modifiers (e.g., silk or gelatin) by enabling low-viscosity polymeric bioinks to be suspended within a self-recovering gel that constrains flow and prevents the eventual collapse of the constructs. The most widely used support baths are either based on a gelatin slurry, commonly referred to as ‘freeform reversible embedding of suspended hydrogels’ (FRESH), or an agarose slurry, originally termed as ‘suspended layer additive manufacturing’ (SLAM) [4,5]. The use of suspension baths carries significant advantages for extrusion bioprinting by improving resolution, increasing cell viability, and enabling the printing of more complex geometries [6]. However, difficulties associated with the removal of the constructs from the supporting bath, restricted control over the crosslinking temperature of the inks (which must be matched to that of the bath), and the impossibility of printing thermoplastic materials still prevent a more widespread use of the technology.

Due to their ability to easily form cell-compatible hydrogels in the presence of divalent cations, alginate-based materials have been widely explored in the formulation of polymeric inks for the encapsulation and printing of mammalian cells. Whilst the biodegradation of alginate is limited and can only be improved through chemical modification (i.e., oxidation) [7,8], bioactivity and printability are generally easier to adjust either by blending it with other materials (e.g., collagen, gelatin) or by varying the polymer concentration and molecular weight [9,10]. To improve structural fidelity and retain cell viability, low-viscosity alginate-based inks can also be printed in suspension or using sacrificial polymers [11,12].

Similarly, hydrogels consisting of nanocellulose, a structural form of cellulose, have shown promising results as a biomaterial suitable for cell encapsulation work, with the previous literature reporting no biocompatibility issues [13,14,15]. Cellulose fibres can be derived from most plants, as well as certain species of algae, tunicates, and bacteria. These fibres can be broken down via mechanical agitation into cellulose microfibrils, which have frequently been used in TE&RM to mimic the structure and organisation of collagen fibres [16,17,18,19,20]. By using a stronger acid hydrolysis treatment, it is also possible to isolate cellulose nanocrystals (CNCs), which can act as liquid crystals if colloidally stabilised. Under specific conditions, chiefly concentration and pH, CNCs can arrange themselves into highly organised and aligned anisotropic phases [21]. The exceptional properties of CNCs, namely their high elastic modulus and strength, have made them an attractive contender as a reinforcing material in TE&RM scaffolds [22,23]. This has been previously reported by other groups who successfully generated stiff composite systems through the addition of high concentrations of up to 15% (*w*/*v*) CNCs to different hydrogel formulations [24,25].

In this work, we investigated the use of commercially available spray-dried CNCs as a rheological enhancer to formulate polymeric inks for bioprinting cell-laden constructs that are suitable for replicating soft tissue environments without compromising on printability, cell distribution, and cell viability. For this purpose, hydrogel blends of alginate and CNCs were created, referred to as AlgCNC, and rheologically characterised to reveal both their shear-thinning capabilities and self-recovery ability following repeated deformation. Upon determining the optimal process parameters, a single AlgCNC formulation was taken forward for encapsulation and bioprinting with immortalised chondrocytes inside an agarose microgel suspension bath. Preliminary in vitro tests based on LIVE/DEAD fluorescent imaging reveal that the viability of cells encapsulated in AlgCNC hydrogels remains high even after printing, confirming the absence of any substantial cytotoxic effects induced by the presence of CNCs or the extrusion process itself.

Overall, the present study provides valuable new insights into the influence of relatively low concentrations of CNCs on 3D polymeric structures constructed using extrusion-based suspension bioprinting. More specifically, this work demonstrates how CNCs can be used to improve the post-crosslinked stability and recovery of soft hydrogel structures, while demonstrating how the preferential alignment of CNCs can be induced using printing parameters and biological conditions that do not compromise cell survival. Collectively, our work shows that CNCs can be employed as an easily accessible additive in the formulation of low-viscosity bioinks to achieve superior printing resolutions for the biofabrication of structurally relevant soft tissue structures.

## 2. Materials and Methods

### 2.1. Materials

Cellulose nanocrystal powder was purchased from CelluForce^©^ (Montreal, QC, Canada) under the commercial name CELLUFORCE NCV100–NASD90. Alginic acid sodium salt from brown algae and agarose (Type I, low EEO), sodium chloride, and calcium chloride dihydrate were purchased from Sigma-Aldrich (Gillingham, UK). All reagents were used without further modifications.

Dulbecco’s modified Eagle’s media (DMEM), foetal bovine serum (FBS), L-glutamine, and TrypLE^™^ Express were purchased from Gibco (Thermo Fisher Scientific, Altrincham, UK). Dulbecco’s phosphate-buffered saline (PBS) without calcium and magnesium was purchased from Sigma-Aldrich (Cambridge, UK). The LIVE/DEAD^™^ Viability/Cytotoxicity kit for mammalian cells was purchased from Invitrogen (Thermo Fisher Scientific, Altrincham, UK).

### 2.2. Preparation of CNC Suspensions

Prior to use, the spray-dried CNCs were homogeneously dispersed in Milli-Q^®^ water (18.2 MΩ.cm) using the following protocol: a 7% (*w*/*v*) CNC suspension was created by gradually adding 3.5 g of CNC powder to 50 mL of deionised water while stirring at a constant speed for 1 h to ensure complete dissolution. An ultra-sonicator probe (Qsonica Q500 Sonicator^®^, Newtown, CT, USA) was employed to create a well-dispersed CNC suspension. The sonication treatment was conducted by centring the probe in the suspension volume and sonicating at 80–100% amplitude in 2 s pulsed intervals for ca. 5 min. The suspension was stored in the fridge until required; the sonication protocol was repeated on the day of use. The particle size of CNCs in suspension was confirmed to be within the optimal range of 60–110 nm using dynamic light scattering (DLS) (Zetasizer Ultra, Malvern Panalytical, UK). In short, 0.1% (*w*/*v*) of CNC suspension prepared in Milli-Q^®^ water was added to 10 mM NaCl solution in a 1:1 ratio and left to stir for 10 min before conducting the measurement.

### 2.3. Preparation of Pre-Gel Formulations

Alginate/CNC (AlgCNC) pre-gel formulations were prepared by adding specific quantities of 4% (*w*/*v*) alginate dropwise to 7% (*w*/*v*) CNC suspension while agitating at ca. 12,000 rpm via magnetic stirring. In addition, either Milli-Q^®^ or PBS was added to the mixture to act as a diluting agent. The two AlgCNC formulations created both consisted of 1% (*w*/*v*) alginate and either 1% (*w*/*v*) or 2% (*w*/*v*) CNCs, labelled Alg1CNC1 and Alg1CNC2, respectively. Alginate (Alg) was prepared at a 1% (*w*/*v*) concentration by diluting 4% (*w*/*v*) alginate in a 1:3 ratio with PBS, which is referred to as Alg1.

### 2.4. Rheological Characterisation

Amplitude, frequency, and time sweeps of alginate and AlgCNC hydrogels were performed using a Discovery Hybrid 2 (DHR-2) rheometer (TA Instruments, New Castle, DE, USA) equipped with a 20 mm parallel plate and 500 μm gap size. Alginate and AlgCNC crosslinked hydrogels were formed by pipetting 400 μL of the pre-gel into a 12-well cell culture insert and placing it in 150 mM CaCl${}_{2}$ for at least 40 min at 37 ${}^{{}^{\circ}}$C to induce ionotropic gelation. Once complete, the gels were removed from the inserts and placed in PBS to wash. Excess PBS was removed by gentle blotting with a tissue before placing it onto the bottom plate of the rheometer for testing. Flow sweeps of alginate and AlgCNC pre-gels were performed using HAAKE MARS iQ Air rheometer (Thermo Fisher Scientific, Waltham, MA, USA) with a 35 mm parallel plate and 1000 μm gap size. For each measurement, 1 mL of pre-gel was pipetted onto the bottom plate. The sample was equilibrated to 37 ${}^{{}^{\circ}}$C (amplitude, frequency, and time sweeps) or 25 ${}^{{}^{\circ}}$C (flow sweeps) for 60 s. The variables for each rheological assessment are shown in Table 1. A minimum of three samples were completed for each rheological test and data are represented as the mean ± standard deviation.

### 2.5. Scanning Electron Microscopy Imaging

Scanning electron microscopy (SEM) was performed using a Zeiss Sigma VP FEG SEM system. For images of CNCs, a 0.01% (*w*/*v*) CNC suspension in deionised water was drop-casted directly onto the SEM substrate and left to dry at room temperature. Prior to imaging, lyophilised AlgCNC hydrogels were cut down the middle with a razor to capture the cross-sectional morphology and then fixed onto 90 degree-angled pin stubs using carbon adhesive tabs. Both samples were coated with an Au/Pt 80:20 target using a 50 mA current for 8 s using a Quorum Q150R Plus sputter-coater.

### 2.6. Preparation of Agarose Suspension Bath

Agarose powder was added gradually to Milli-Q^®^ water whilst stirring to make a 0.5% (*w*/*v*) agarose gel, which was autoclaved at 121 ${}^{{}^{\circ}}$C, 1.4 bar pressure. The resulting solution was then cooled to room temperature whilst being subjected to a constant shear force via magnetic stirring at ca. 1000 rpm.

### 2.7. Printing Optimisation

The printability of alginate-based inks was evaluated using a 3D Discovery^™^ bioprinter (RegenHU, Villaz-St-Pierre, Switzerland) within a biosafety cabinet equipped with a pneumatic extrusion printhead and 25 gauge blunt-end straight needle. Alg1 or Alg1CNC1 pre-gel formulations, each containing red food colouring to aid visualisation, were transferred into 3 mL printing cartridges with a piston. BioCAD^™^ software was used to design single-layered horizontal lines which were then coded (i.e., G-code) and printed directly inside a 6-well tissue culture plate containing a suspension of agarose fluid gel. Applied air pressure to extrude the ink was varied between 0.018 MPa and 0.050 MPa for printing feed rates of 10, 35, 50, and 60 mm s^−1^. Images were taken from above the print using a Dino-Lite USB digital microscope and were imported into ImageJ software (National Institute of Health, Bethesda, MA, USA; version 1.52e) to measure filament diameters from six different areas. The resultant values are reported as their mean ± standard deviation (SD).

### 2.8. Chondrocyte Culture and Encapsulation in AlgCNC Pre-Gels

Human-derived TC28a2 immortalised chondrocytes were cultured in DMEM containing 10% FBS and 2 mM L-glutamine and maintained at 37 ${}^{{}^{\circ}}$C, 5% CO_2_. To prepare for AlgCNC and Alg encapsulation, cells at ca. 80% confluence were trypsinised and counted, before aliquoting at the required cell density and pelleting by centrifugation at 600 g for 5 min. AlgCNC and Alg pre-gels were sterilised using an autoclave at 121 ${}^{{}^{\circ}}$C and 1.4 bar. For cell-laden hydrogels, sterile pre-gel was dispensed and mixed with pelleted cells (1 × $10^{6}$ cells/mL) using a positive displacement pipette until homogeneous. The resultant cell-laden pre-gel was transferred into 12-well plate cell culture inserts (250 μL per insert) and crosslinked using 150 mM CaCl_2_ dissolved in DMEM for 1 h, before replacement with cell culture media. The cell-laden AlgCNC hydrogels were incubated at 37 ${}^{{}^{\circ}}$C and 5% CO_2_.

### 2.9. 3D Bioprinting of Cell-Laden Hydrogels

The bioprinting of cell-laden Alg1CNC1 and Alg1 pre-gel suspensions was conducted as described in Section 2.7. Immortalised chondrocytes were encapsulated within the pre-gel suspensions at a density of 2 × $10^{6}$ cells/mL. Single-layer grid structures of Alg1 or Alg1CNC1 were printed into an agarose suspension bath inside a 12-well plate with a feed rate of 10 or 60 mm s^−1^ and an applied pressure of 0.020 or 0.030 MPa, respectively. Pre-gel printed structures were crosslinked with 150 mM CaCl_2_ dispensed directly on top of the agarose bath, followed by incubation at 37 ${}^{{}^{\circ}}$C and 5% CO_2_ for ca. 1 h to allow for the diffusion and crosslinking of printed gels. Fully crosslinked gels were then removed from the agarose and transferred to a fresh 12-well cell culture plate containing cell culture media and were incubated at 37 ${}^{{}^{\circ}}$C and 5% CO_2_.

### 2.10. LIVE/DEAD Assay

The biocompatibility of AlgCNC cell-laden hydrogels was assessed using a LIVE/ DEAD assay at 24 h post-manual cell encapsulation and post-bioprinting, as well as 7 days post-manual cell encapsulation. Calcein-AM (live) and ethidium homodimer-1 (dead) markers were used at concentrations of 2 μM and 4 μM in PBS, respectively. The gels were incubated with the markers for 20 min at 37 ${}^{{}^{\circ}}$C, before imaging with an optical fluorescent microscope. Images were processed and cell numbers were quantified using ImageJ software (National Institutes of Health, USA; version 1.52e). Cell viability was calculated by taking the number of live cells and dividing it by the total number of cells, averaged over three images for each gel.

### 2.11. Data and Statistical Analysis

Data points on graphs were created using GraphPad Prism (GraphPad Software, LLC; version 9.1.2 (226)) and are displayed as mean averages with error bars representing standard deviation. GraphPad Prism was also used to perform a two-way ANOVA with the Tukey comparison test for the rheological flow sweep data, with *p* ≤ 0.05 considered statistically significant, and **** *p*≤ 0.0001.

## 3. Results and Discussion

### 3.1. Characterisation of Cellulose Nanocrystals

**Figure 1:**
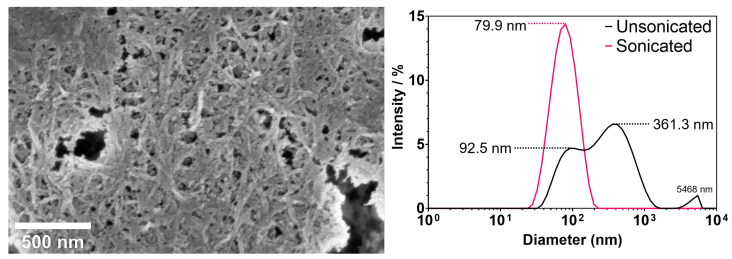
SEM image of sonicated CNCs drop-casted directly onto the sample holder as a 0.01% suspension in deionised water (**left**). DLS analysis to characterise CNC size distributions before and after sonication treatment; maximum diameters are labelled for each peak maximum (**right**).

The particle size distribution of cellulose nanocrystals (CNCs) suspended in deionised water was measured to confirm a homogeneous dispersion of nanoparticles throughout the suspension (Figure 1 (right)). Data collected using dynamic light scattering (DLS) confirmed a narrow size distribution for CNC suspensions that had undergone ultrasonication treatment, showing a peak maximum at 79.9 nm. Unsonicated CNC suspensions showed three distinct particle size maximum peaks and a wide particle distribution, suggesting the presence of CNC aggregation and inhomogeneity. It is important to note that, while DLS can provide reliable information on the homogeneity of the suspension, it can only provide a rough estimate on the apparent CNC particle size due to their non-spherical morphology [26].

Images of CNCs were captured using scanning electron microscopy (SEM) using a low concentration suspension, which revealed the presence of random crystal alignment with uniform needle-like morphologies (Figure 1 (left)).

### 3.2. Rheological Characterisation of AlgCNC Formulations

The rheological properties of AlgCNC formulations were examined as crosslinked hydrogels and as pre-gels (i.e., not crosslinked). Understanding the rheological response of the pre-gels to an applied shear is important to assess the potential printability of the materials. Ideally, a polymeric ink or bioink should exhibit a high enough viscosity to ensure the proper formation of a filament upon extrusion through a needle, and to prevent the sedimentation of encapsulated cells within the printing cartridge. However, too high a viscosity can have a detrimental effect on cell viability due to the higher shear stresses generated during the extrusion process of the bioinks. Hence, it is desirable for bioinks to exhibit non-Newtonian properties, specifically shear-thinning behaviour, which causes a drop in viscosity when passing through the narrow printing needle. Additionally, the flow response of the pre-gel on applying shear forces can have a major impact on its printing resolution, while it has also been shown to induce the alignment of suspended particles [27,28,29].

**Figure 2:**
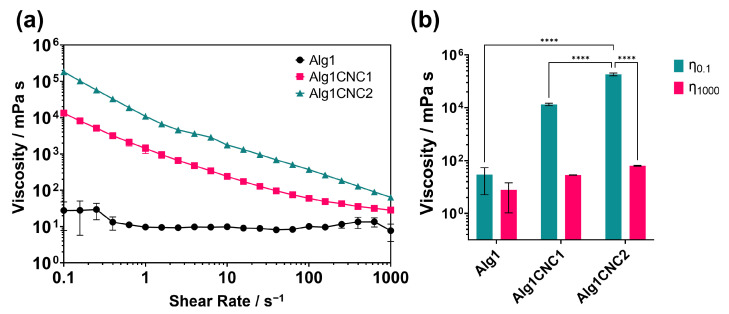
(**a**) Flow sweep experiments showing the viscosity response of Alg1 (black), Alg1CNC1 (pink), and Alg1CNC2 (green) to an increasing shear rate. (**b**) Comparison of viscosity values for Alg1, Alg1CNC1, and Alg1CNC2 at a shear rate of 0.1 s^−1^ (green) and 1000 s^−1^ (η1000, pink). (**** *p* < 0.0001).

Flow sweep tests were performed to assess the effect on the viscosity when shear forces are applied to pre-gel formulations of Alg1CNC1 and Alg1CNC2, containing 1% (*w*/*v*) CNCs and 2% (*w*/*v*) CNCs, respectively. Alg1 was used as a control for all experiments (Figure 2).

Based on Figure 2, it is apparent that the concentration of CNCs has a dominating effect on the viscosity of the pre-gel. Both Alg1CNC1 and Alg1CNC2 exhibit significantly higher viscosities at low shear rates in comparison with Alg1—approximately 10^3^ and 10^4^ times more for each respective formulation. On applying shear forces, Alg1CNC1 and Alg1CNC2 both show shear-thinning behaviour (i.e., decreasing viscosity with increasing shear rate), eventually reaching a similar viscosity to Alg1 at the maximum shear rate measured (Figure 2b). In contrast, Alg1 displays classic Newtonian behaviour with constant viscosity values over the range of tested shear rates. Interestingly, Alg1CNC1 exhibits a plateau region at higher shear rates, which is likely due to CNC orientation reaching its maximum degree along the direction of the applied shear, as previously reported [30,31,32]. It could be hypothesised that more shear force would be required for this effect to occur with Alg1CNC2; thus, this plateau would be expected to be present at a shear rate higher than the 1000 s^−1^ applied in this test.

Similar shear-thinning flow profiles have also been validated to occur in other nanocomposite systems that employ nanosilicates and ceramic-based nanoparticles; however, both have been shown to be osteoconductive, which reduces their capacity to be used as universal additives for tissue bioprinting [33,34,35,36]. Furthermore, Markstedt et al. demonstrated the shear-thinning capacity of cellulose nanofibrils (CNF) when combined with low concentrations of alginate [37]. Derived from the same source, CNFs differ to CNCs in terms of their lower aspect ratio and ribbon-like morphology. As such, their ability to entangle with one another gives rise to a gel-like consistency. Consequently, alginate/CNF gels at similar concentrations to the alginate/CNC concentrations used in this study possess viscosities that are 10^3^ times greater in magnitude. When compared to CNCs, CNFs require higher printing pressures to extrude the material through a nozzle, which may increase the probability of shear-induced cell death, while also necessitating larger nozzle gauges, resulting in reduced printing resolutions. However, their higher resting viscosity denotes that CNF-based bioinks can be printed without the need for a supporting bath.

**Figure 3:**
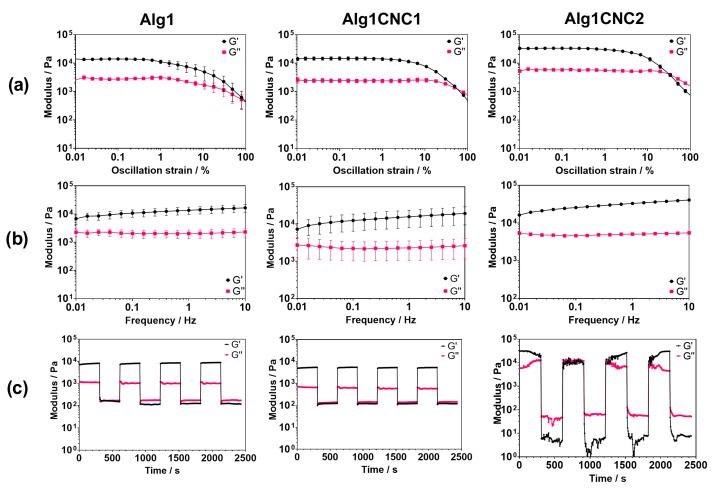
Oscillatory rheological experiments to determine the storage (G’) (black) and loss (G”) (pink) moduli of crosslinked Alg1, Alg1CNC1, and Alg1CNC2 over a range of conditions. (**a**) Amplitude sweeps conducted at a frequency of 1 Hz. (**b**) Frequency sweeps conducted at an oscillation strain of 0.1%. (**c**) Time sweeps conducted at an oscillation strain of 0.1% for 300 s followed by 100% strain for 300 s at constant frequency of 1 Hz.

The same formulations were subjected to oscillatory rheological tests after being fully crosslinked using calcium chloride (Figure 3). All experiments were conducted at 37 ${}^{{}^{\circ}}$C to emulate physiological conditions.

In addition to understanding the flow properties of a bioink, it is crucial to probe the viscoelastic properties of the fully-crosslinked hydrogel to evaluate its capacity to withstand mechanical loads whilst deforming elastically without losing structural integrity. This information is particularly relevant when attempting to mimic the properties of load-bearing tissues such as bone or cartilage. For the latter, hydrogels should be able to remain stable under repetitive shear loading cycles, with no indication of stiffening or softening, thus bearing long-term durability in an environment that is susceptible to repeated movement and deformation. Additionally, the mechanical characteristics of the cell’s surrounding environment are known to have an impact on cellular behaviour, with cells having to exert more force against a stiffer material in remodelling their local environment [38]. Accordingly, this can influence not only cell viability and phenotype but also the overall structural organisation and function of the neotissue [39,40,41].

Amplitude sweeps, shown in Figure 3a, reveal that all formulations exhibit characteristic viscoelastic behaviour, with an initial independence in the storage (G’) and loss (G”) moduli to increasing strain, known as a linear viscoelastic region (LVR). The results indicate that CNCs expand the LVR and consequently the range of strains that can be applied to the hydrogel before micro-cracks begin to form. This suggests the presence of entanglements between alginate chains and CNCs. However, AlgCNC formulations require lower oscillation strains before reaching a yield point (G’ = G”) compared to Alg1, which reaches its yield point only at 100% oscillation strain.

Frequency sweeps were conducted within the LVR of all three formulations at 0.1% oscillation strain. The results confirmed viscoelastic gel behaviour with a clear independence between G’, G” and frequency, as illustrated in Figure 3b. Notably, all three formulations show a consistent 5 kPa difference between G’ and G” across the tested frequencies. These results also confirmed that the inclusion of 1% (*w*/*v*) CNCs to alginate has a limited effect on the measured storage modulus, with a difference of around 2 kPa between Alg1 and Alg1CNC1 at 1 Hz. However, at a concentration of 2% (*w*/*v*) of CNCs, the storage modulus of Alg1CNC2 measures close to 2.5 times larger than Alg1.

Time sweeps were performed to assess how the hydrogels recover after set periods of high oscillation strains (Figure 3c). The addition of CNCs (up to 1% (*w*/*v*)) does not seem to affect the performance of the polymeric systems, with both Alg1 and Alg1CNC1 displaying similar recovery profiles of G’ and G” after each high oscillation strain. In contrast, Alg1CNC2 does not show rapid recovery of G’ and G” after high-strain deformation, instead taking almost the full 2400 s to return to its original moduli. This CNC concentration-dependent recovery was also reported in the work of Fazilati et al. and Huang et al., in which low-concentration CNC suspensions, partly consisting of CNC tactoid assemblies, exhibited slower recovery of their original rheological properties after the application of a high strain compared to those featuring highly arranged liquid crystal CNC suspensions [42,43]. The formation of CNC tactoids is also concentration-dependent; below an environmentally-specific concentration, CNCs are arranged in an isotropic manner. Therefore, we hypothesise that the slow recovery of Alg1CNC2 may be due to CNC self-assembled moieties being dissociated by the applied high stress, which then slowly reassemble into nematic entities over time. The results may also suggest that CNCs within Alg1CNC1 are arranged isotropically. Nonetheless, further experiments would be required to confirm this hypothesis.

While results suggest that both Alg1CNC1 and Alg1CNC2 have similar stabilities over a range of frequencies, the inclusion of CNCs in alginate minimally reduces the oscillation strain required to break the gel. This may be due to CNCs disrupting the integrity of the crosslinked alginate network. Despite this, Alg1CNC1 displays an exceptional recovery of its structural properties following repeated high deformations while maintaining its integrity against higher shear stresses. Due to its slow recovery after repeated deformations, Alg1CNC2 was not carried forward for the following experiments.

### 3.3. Optimisation of Printing Parameters

**Figure 4:**
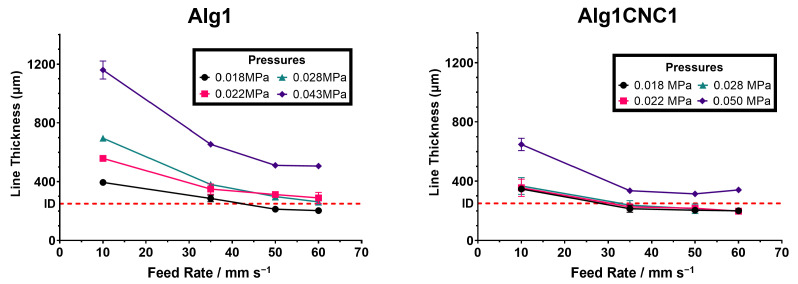
Printing optimisation data for Alg1 and Alg1CNC1 comparing the programmed feed rate versus the measured print line thickness for multiple applied pressures (shown in the respective legends). I.D = internal diameter of the printing needle; I.D = 250 μm.

The ability to physically recreate a digital model with high shape fidelity and reproducibility depends largely on the correct choice of processing parameters. For that purpose, a simple digital model characterised by an array of six 10 mm parallel lines separated by a 2 mm distance was initially created in BioCAD^™^ software. Through a series of trials, the models were then physically reproduced by varying iteratively one parameter (e.g., pressure) while maintaining the other one constant (e.g., feed rate). Images were taken following the completion of each print using a USB camera microscope (displayed in Figures S1 and S2); they were imported onto ImageJ to measure the diameter of the printed filaments. Printing accuracy was determined by comparing the diameter (i.e., thickness) of the printed filaments against the internal diameter of the needle and using the smallest differences between the two as an indicator of higher accuracy and shape fidelity (Figure 4). The maximum feed rate was constrained to 60 mm s^−1^ due to the disturbance of the suspension bath from rapid printer movements above this rate, which in turn adversely affected the print resolution.

Overall, Alg1CNC1 achieved superior printing accuracy for each tested variable when compared to Alg1. Despite its higher viscosity, Alg1CNC1 can be printed at relatively low pressures due to its shear thinning properties. On removing the shear stress, i.e., post-extrusion, Alg1CNC1 returns to its higher at-rest viscosity, resulting in smaller filament diameters compared to the less viscous, hence more spreadable, Alg1. Additionally, Alg1CNC1 can form filaments with dimensions equal to that of the internal diameter of the printing needle and at lower feed rates and pressures to that of Alg1. This is of importance due to the detrimental impact that high shear stresses, caused by increasing extrusion pressures and feed rates, have on the viability of encapsulated cells [44,45]. Printing within a suspension bath, instead of in air onto a printing platform, enhances the resolution of the printed constructs by restricting the flow of the extruded material. As an example, by using a feed rate of 35 mm s^−1^ and pressure of 0.020 MPa, Alg1CNC1 can achieve a filament diameter of around 205 μm–45 μm less than that of the printing needle internal diameter. This is particularly useful for printing constructs with fine details without sacrificing cell viability by using higher pressures and printing speeds. The highest pressures shown Figure 4 for Alg1CNC1 and Alg1 define the limiting pressures at which it is no longer possible to achieve a line thickness close to that of the internal diameter of the needle.

It is important to emphasise that these results cannot be directly applied for bioprinting cell-laden structures, and that small adjustments in pressure and/or feed rate may be necessary to compensate for the increase in viscosity caused by the addition of cells. Instead, they can act as estimations to select appropriate variables before making suitable adjustments when executing the bioprinting process. Nonetheless, these results can clearly support the inclusion of CNCs within low-viscosity hydrogels to improve their printing resolutions. This allows for more accurate recreations of biologically relevant constructs, thus improving the integration with the native environment in implantation or in creating representative disease models.

### 3.4. Morphological Characterisation of AlgCNC

**Figure 5:**
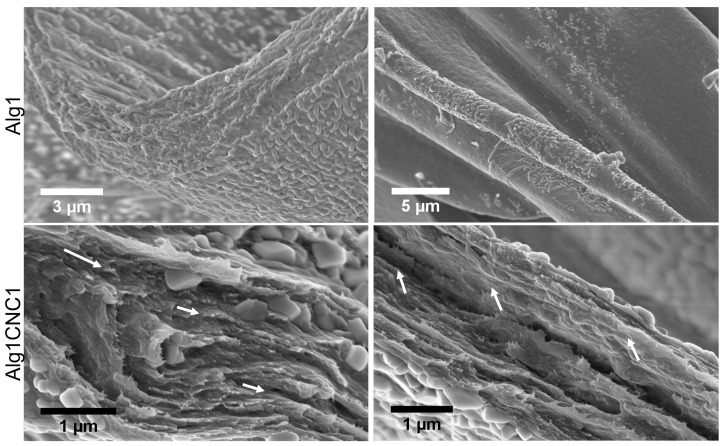
SEM images of Alg1 and AlgCNC1 filaments formed via extrusion-based 3D printing. Images taken show the cross-sections of the filaments from lyophilised 3D printed constructs at magnifications of (from top left to bottom right) 45k×, 25k×, 162k×, 133k×. Arrows indicate the preferential local directionality of CNCs.

Scanning electron microscopy (SEM) was used to image the internal morphology of 3D-printed filaments of Alg1CNC1 versus Alg1 (Figure 5). More specifically, the images were used to examine the organisation of CNCs within the alginate network after being extruded through the printing needle.

At lower magnifications, the internal architectures of Alg1 and Alg1CNC1 were comparable with few identifiable differences. The surfaces were largely coated in calcium chloride salt deposits from the crosslinking process, yet surfaces not coated in salt appeared smooth. Increasing the magnification at cross-sections revealed an even distribution of CNCs throughout the construct. CNCs also appear to induce the formation of distinct layers with some evidence of preferential orientation, as indicated by the white arrows in Figure 5. Both of these findings suggest that the existence of anisotropic CNC self-assembly is possible within an alginate network. We hypothesise that the CNC alignment seen in the SEM images was induced by the shear forces created during extrusion through the printing needle; however, lyophilising the soft printed materials to prepare for imaging caused a structural collapse that made the distinctive filament lines ambiguous. Previous work by Esmaeili et al. and Hausmann et al. have extensively characterised CNC alignment during extrusion by exploiting the birefringent nature of CNCs and imaging their orientation in situ using optical polarised microscopy [32,46]. Their findings showed that CNC alignment could be induced and controlled by multiple factors, including pressure/shear rate and nozzle diameter.

Aligned CNCs within printed AlgCNC filaments may be able to be used as templates to guide the deposition and formation of fibrillar structures by mammalian cells, such as collagen fibrils in articular cartilage. Favourable interactions between negatively charged CNCs and collagen have previously been utilised to make collagen-based hydrogels [47,48]. Therefore, we speculate that these interactions could be exploited by shear-aligning CNCs via extrusion-based bioprinting to create an accurate representation of the depth-dependent fibrillar arrangements found in native cartilage tissue.

### 3.5. Cell Viability of AlgCNC Hydrogels

A preliminary evaluation of the biocompatibility of AlgCNC hydrogels was conducted by manually encapsulating TC28a2 immortalised chondrocytes within Alg1CNC1 and Alg1 and monitoring their cell viability over the course of a week. Additionally, the chondrocytes were encapsulated in Alg1 and AlgCNC1 and extruded through a 250 μm needle directly inside a suspension bath of agarose.

**Figure 6:**
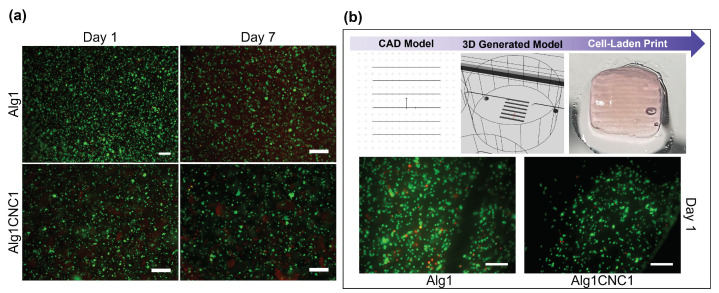
(**a**) Fluorescence microscopy images of immortalised TC28a2 chondrocytes 1 and 7 days post-manual encapsulation within Alg1 and Alg1CNC1 hydrogels; (**b**) Top row: general workflow for 3D bioprinting of cell-laden constructs. Bottom row: fluorescence microscopy images of TC28a2 chondrocytes within Alg1CNC1 and Alg1 captured 1 day post-bioprinting. Images taken using a LIVE/DEAD assay to distinguish between viable cells (green) and dead cells (red). Scale bar represents 200 μm.

LIVE/DEAD assays after one and seven days of culture in the hydrogels show that the viability for manually encapsulated cells within both formulations dropped slightly throughout the course of the experiment (Figure 6a). Indeed, some cell death was present at all time points, although this was to be expected due to the somewhat disruptive cell encapsulation process. Despite this, image analysis showed that cell viability remained above 70% in the first seven days after encapsulation. Importantly, Alg1CNC1 maintained similar cell viability levels to Alg1 through the time course. This finding may support the argument that a loss of cell viability over time is due to the cell encapsulation process, which requires repeated pipetting to homogeneously combine the pre-gel with the cells, and not the presence of CNCs themselves. In particular, Alg1CNC1 pre-gels required more vigorous pipetting to create homogeneous suspensions compared to Alg1 due to their higher viscosity, although the shear-thinning properties facilitated this procedure.

Additionally, images in Figure 6a taken at seven days post-encapsulation also reveal that a spherical cell morphology is maintained throughout the culture period in both formulations. Preserving a spherical shape and preventing the elongation of chondrocytes is known to have implications on collagen type II production, which is a ubiquitous component of articular cartilage [49,50,51].

The printing parameters were established based on the results from Section 3.3, using the lowest possible pressure to obtain a filament diameter similar to that of the internal diameter of the printing needle. As expected, adjustments to the printing parameters were required due to the rheological changes of the pre-gels upon the addition of cells. In the case of Alg1CNC1, both the pressure and print speed were increased due to the apparent heightened viscosity and shear-thinning properties of the formulations, respectively.

Quantitative analysis of fluorescence images using ImageJ shown in Figure 6b revealed an estimated 75% and 94% cell viability for bioprinted Alg1 and Alg1CNC1, respectively. Similar to manually encapsulated gels, bioprinted cells also retained their spherical morphology post-extrusion, aligning with the objective of preventing cell elongation to avoid fibrocartilage formation. Additionally, cells were seen to stay within the bounds of their printed filaments, thus maintaining their intended organisation. Importantly, cells encapsulated within Alg1CNC1 were homogeneously spread throughout the whole printed constructs, while the densities of cells within Alg1 were not as consistent throughout the print. This supports the rationale for incorporating CNCs into alginate with the aim of reducing the cell sedimentation occurring within the printing cartridge, or even preventing it. Taken together, these results suggest that the combination of pressure-assisted extrusion and SLAM bioprinting can be effectively used with TC28a2 cells without affecting cell viability.

## 4. Conclusions

Here, we have demonstrated how CNCs can be effectively incorporated in low-viscosity alginate-based hydrogels to formulate bioinks with improved rheological properties for the bioprinting of cell-laden constructs with high cell viability. Rheological tests showed that the addition of CNCs imparts strong shear-thinning properties onto alginate solutions, which is beneficial for preventing needle clogging to facilitate flow during extrusion-based 3D printing. Additionally, CNCs can extend the limit of applied strain before yielding begins to occur (LVR), while improving printing resolution when using relatively low pressures and printing speed, which is recommended for increasing the probability of cell survival. Alg1CNC1 formulations showed good biocompatibility using encapsulated chondrocytes, which was also reciprocated in bioprinted formulations, confirming that the printing process did not detrimentally affect cell survival. At the nanoscopic level, CNCs have been shown to be uniformly distributed with preferential directionality within the alginate network.

Overall, our data suggest that CNCs can be used as an affordable and easily accessible additive for incorporation into base polymer hydrogel formulations to augment its rheological characteristics without significantly affecting the overall strength of the hydrogel. In particular, when used in small concentrations of up to 1% (*w*/*v*), CNCs can contribute to improve printability, mechanical stability, and to prevent cells from settling within the printing cartridge. Alginate is unable to support cellular development due to a lack of cell surface receptors, while also lacking in vivo biodegradability within the human body. Despite this, we foresee the use of CNCs alongside more progressive hydrogel systems, such as those employing light-based crosslinking and growth factor delivery systems, to create bioengineered scaffolds that can effectively reproduce the complex collagenous organisations for superior tissue repair and disease modelling applications.

## Figures and Tables

**Table 1:** Variables used for amplitude, frequency, and time sweeps of AlgCNC hydrogels and flow sweeps of AlgCNC pre-gels.

	Oscillation Strain	Frequency	Shear Rate
**Amplitude Sweep**	0.01–100%	1 Hz	-
**Frequency Sweep**	0.1%	0.001–10 Hz	-
**Time Sweep**	0.1% (300 s); 100% (300 s)	1 Hz	-
**Flow Sweep**	-	-	0.1–1000 s${}^{-1}$

## Data Availability

Data are contained within this article or Supplementary Materials.

## Supplementary Materials

The following supporting information can be downloaded at https://www.mdpi.com/article/10.3390/pharmaceutics15102432/s1, Figure S1: Images taken using a USB camera microscope of completed prints using Alg1 dyed with red food colouring to aid visualisation. Pressures and feed rates were varied to determine their effect on the printing accuracy; Figure S2: Images taken using a USB camera microscope of completed prints using Alg1CNC1 dyed with red food colouring to aid visualisation. Pressures and feed rates were varied to determine their effect on the printing accuracy.

## Abbreviations

The following abbreviations are used in this manuscript:

3D	Three-dimensional
AM	Additive manufacturing
Alg	Alginate
AlgCNC	Alginate/cellulose nanocrystal formulation
CNC	Cellulose nanocrystals
CNF	Cellulose nanofibrils
DLS	Dynamic light scattering
DMEM	Dulbecco’s Modified Eagle Medium
ECM	Extracellular matrix
FRESH	Freeform Reversible Embedding of Suspended Hydrogels
LVR	Linear viscoelastic region
OA	Osteoarthritis
SLAM	Suspended Layer Additive Manufacturing
SEM	Scanning Electron Microscopy
TE&RM	Tissue engineering and regenerative medicine
