# Supplementary material for: Nanocrystalline Cellulose as a Versatile Engineering Material for Extrusion-Based Bioprinting
Source: Pharmaceutics. 2023 Oct 7;15(10):2432. doi: 10.3390/pharmaceutics15102432 (PMC10609932; doi:10.3390/pharmaceutics15102432)
Supplement: Supplementary file 1 [file pharmaceutics-15-02432-s001.zip › MDPI_AlgCNC_Supplementary_Material.pdf]

Nanocrystalline Cellulose as a Versatile Engineering Material for Extrusion-Based Bioprinting

Supplementary Material

Sophia A. Read, Chee Shuen Go, Miguel J.S. Ferreira, Cosimo Ligorio, Susan J. Kimber, Ahu G. Dumanli and Marco A. N. Domingos

S.1 Printing Optimisation

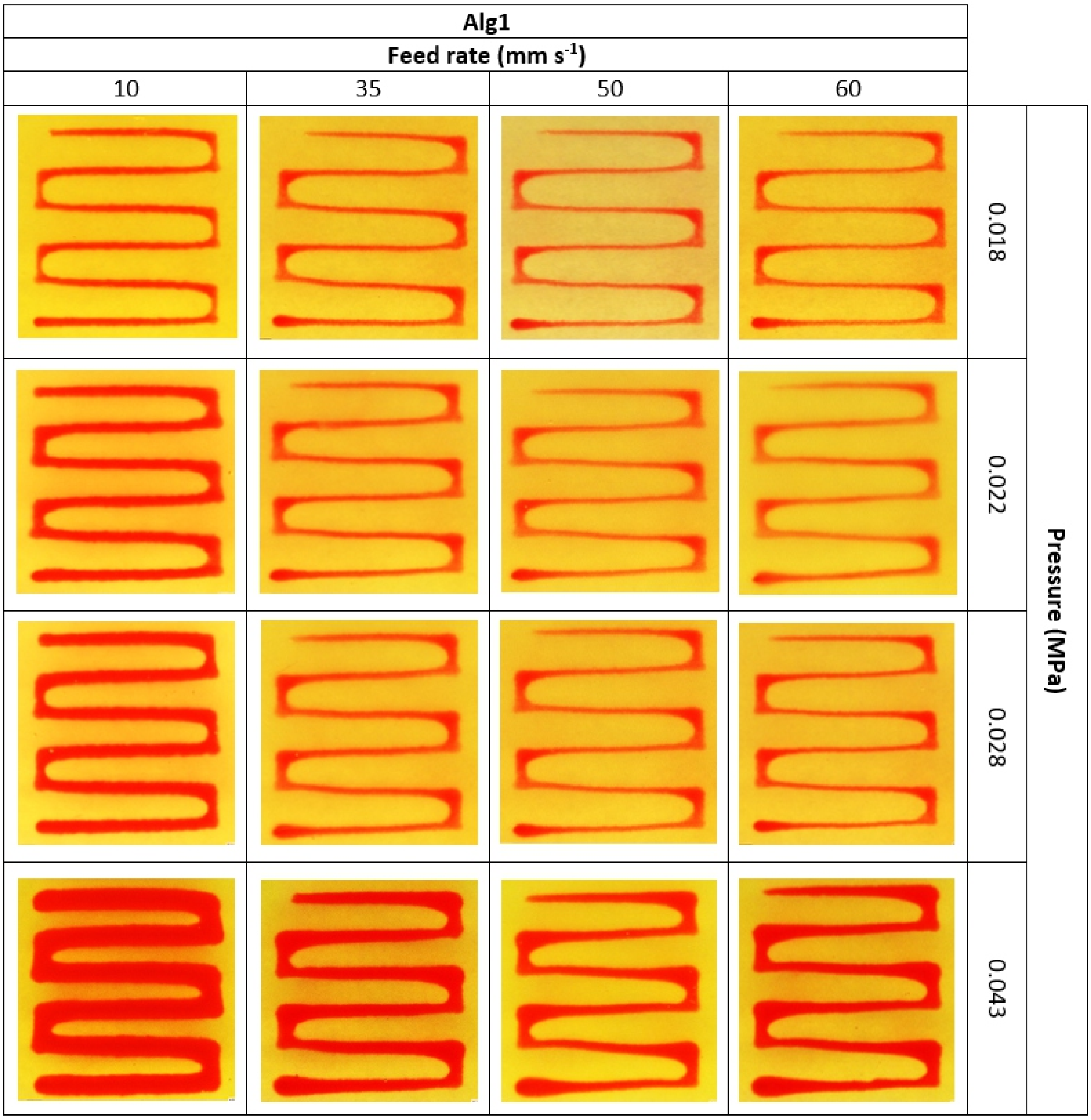

Figure S1: Images taken using a USB camera microscope of completed prints using Alg1 dyed with red food colouring to aid visualisation. Pressures and feed rates were varied to determine their effect on the printing accuracy.

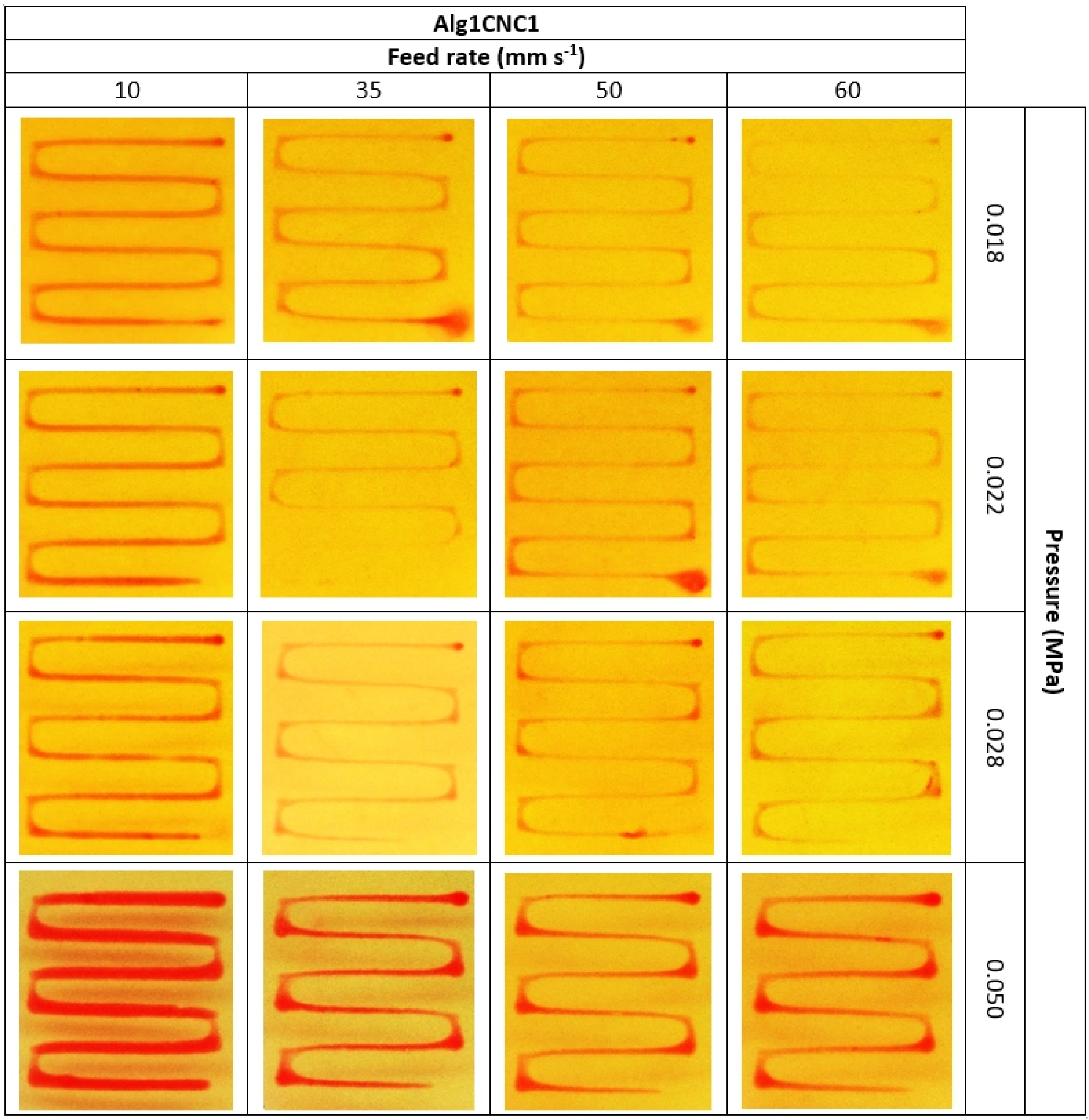

Figure S2: Images taken using a USB camera microscope of completed prints using Alg1CNC1 dyed with red food colouring to aid visualisation. Pressures and feed rates were varied to determine their effect on the printing accuracy.
